# Global and regional burden of nasopharyngeal cancer in older adults attributable to smoking and high alcohol use from 1990 to 2021

**DOI:** 10.3389/fpubh.2025.1614389

**Published:** 2025-06-12

**Authors:** Xiao Wang, Ying Li, Yujie Luo, Xin Song

**Affiliations:** ^1^Department of Otorhinolaryngology, Head and Neck Surgery, Ziyang Central Hospital, Ziyang, China; ^2^Department of Ophthalmology, Ziyang Central Hospital, Ziyang, China

**Keywords:** healthy aging, nasopharyngeal cancer, smoking use, alcohol use, GBD

## Abstract

**Background:**

Nasopharyngeal cancer (NPC) poses a considerable global health burden, with behavioral risk factors such as smoking and high alcohol use contributing to disparities across sociodemographic groups. The growing aging population faces heightened vulnerability to NPC due to prolonged exposure to these modifiable risks, yet comprehensive analyses of aging-specific burden patterns remain limited.

**Methods:**

Utilizing the Global Burden of Disease 2021 data, we conducted a systematic evaluation of NPC burden attributable to smoking and alcohol use across 204 countries from 1990 to 2021. Age-stratified analyses focused on older adults (≥60 years), incorporating three analytical dimensions: Sociodemographic Index (SDI) quintiles, sex-specific disparities, and geospatial heterogeneity. Age-standardized mortality (ASMR) and age-standardized DALY rates (ASDRs) were calculated with 95% uncertainty intervals. Temporal trends were quantified via estimated annual percentage changes (EAPCs). Bayesian age-period-cohort modeling projected disease burden through 2050.

**Results:**

From 1990 to 2021, smoking and high alcohol use contributed substantially to the NPC burden globally, with older adults exhibiting distinct risk profiles. Decline in smoking-related burden in global ASDR was observed, yet older adults in low and middle SDI regions retained disproportionately high rates. Rise in alcohol-related burden in certain regions (e.g., Southeast Asia and Caribbean) was particularly pronounced among older age groups. Middle and high-middle SDI regions consistently exhibited the highest ASDR for both risk factors, with older adults contributing a significant share of disability-adjusted life years (DALYs). Older males faced the highest DALY burdens, with extreme older male-to-female disparities persisting across age groups. Population aging will amplify absolute DALY burdens among older adults by 2050.

**Conclusion:**

Behavioral risk factors such as tobacco and alcohol use remain key contributors to the burden of nasopharyngeal cancer in older persons, with significant regional, gender, and demographic differences. There is an urgent need to develop targeted public health policies focusing on smoking cessation and alcohol control that take into account the health needs of older persons.

## 1 Introduction

Nasopharyngeal carcinoma (NPC) is a malignant tumor originating from the epithelial cells of the nasopharyngeal mucosa with significant geographic distribution differences and racial predisposition (1). Despite its low overall incidence globally, NPC exhibits a highly concentrated prevalence pattern in specific populations and geographic regions (2, 3). According to the International Agency for Research on Cancer (IARC), in 2020, there were approximately 133,000 new cases of NPC worldwide, resulting in more than 80,000 new cases of NPC (4, 5). The etiology of NPC is complex and involves the interaction of multiple environmental, genetic, and viral factors. Among them, Epstein-Barr virus (EBV) infection is widely recognized as one of the core factors in its pathogenesis (6). In addition, dietary factors (e.g., high intake of preserved foods), cigarette smoking, air pollution, occupational exposures, and genetic predisposition have been shown to be closely associated with NPC (7–9). Emerging evidence underscores that older adults (≥60 years) are uniquely vulnerable to NPC due to lifelong cumulative exposure to these risks, compounded by age-related declines in immune function and healthcare access disparities. Despite global aging trends, systematic analyses of NPC burden patterns in older populations, particularly in low- and middle-income countries (LMICs), remain scarce, limiting targeted prevention strategies for healthy aging (10). Prior Global Burden of Disease Database (GBD) studies have mapped global NPC trends but lacked age-stratified analyses for older adults. Regional investigations in LMICs focused predominantly on younger cohorts or single risk factors (e.g., EBV). In contrast, this study is the first to integrate smoking and alcohol use as dual behavioral drivers of NPC burden in older adults (≥60 years) across 21 GBD regions, enabling a granular examination of aging-specific disparities and risk synergies. This approach addresses critical gaps in understanding how population aging intersects with modifiable behaviors to shape NPC epidemiology (11).

Based on previous GBD studies, smoking and alcohol abuse have been identified as major modifiable risk factors for a number of cancers, including NPC (11). Despite global efforts to control tobacco and increase awareness of alcohol-related health risks, the spatial and temporal patterns of the burden of NPC due to these two behavioral risks have not yet been comprehensively examined over time at the global, regional, and national levels (12). Understanding these temporal trends is essential to inform targeted public health interventions and resource allocation, especially in high-prevalence areas (13).

This study assesses the burden of NPC attributable to smoking and high alcohol use from 1990 to 2021, with a focus on aging-specific health impacts. We analyze ASDR, EAPC, and SDI disparities across sex and age strata (emphasizing ≥60 years). Projections to 2050 evaluate how population aging interacts with behavioral risks to shape future disease burden, aiming to inform equity-oriented interventions for older adults (14, 15). By comprehensively characterizing the long-term impact of behavioral risk factors on the NPC disease burden, aiming to fill the research gap in the current literature, we expect to reveal the dynamic evolutionary characteristics of the global disease burden of NPC and provide evidence-based support for countries to develop targeted cancer prevention and control strategies (5, 16).

## 2 Methods

Data on NPC burden attributable to smoking and high alcohol use were obtained from the GBD 2021 study, covering the years 1990 to 2021 across 21 GBD regions and five SDI categories. Stratification analyses were stratified by sex (older males and females), global region, and SDI quintile (low to high SDI). SDI is a composite measure reflecting income, education, and fertility. Key metrics included ASMR, ASDR, and EAPC. NPC cases attributable to each risk factor were identified using the comparative risk assessment framework.

EAPC was calculated to evaluate temporal trends in ASMR and ASDR, and sex ratios were used to assess gender disparities. Analyses were stratified by sex, region, and SDI quintiles (low, low-middle, middle, high-middle, and high SDI). All analyses were conducted using publicly available data and standardized GBD methodologies to ensure consistency and comparability (17–19).

DALYs are a central measure of the overall burden of disease. It is the sum of healthy life years lost due to premature death and disease and is used to reflect the overall impact of a health problem on an individual or population.


$$\begin{array}{l} DALYs=\text{YLL }+\text{ YLD} \end{array}$$

YLL, Loss of life expectancy due to premature death; YLD, Non-fatal disability burden of disease.

To mitigate variations in population age structures across regions, ASRs were employed to assess the burden of NPC across different time periods, genders, and geographic locations. The ASR was calculated using the following formula:


$$\begin{array}{l} ASR=\frac{\sum_{i=1}^{N}\;\left(\alpha_{i}\times W_{i}\right)}{\sum_{i=1}^{N}\;W_{i}} \end{array}$$

In the age-specific crude rate for the i-th age group, *W*_*i*_: population weight (or count) of the i-th age group in the GBD standard population, and N: total number of age groups. The 25th and 975th values of the ordered draws were selected as the lower and upper bounds of the 95% Uncertainty Interval (UI), respectively.

Trend analysis EAPC was calculated using a log-linear regression model, with trends over time assessed through the slope of the regression line of the log-transformed rates. To assess temporal trends in ASMR and ASDR, we applied a log-linear regression model to calculate the EAPC. The model is defined as:


$$\begin{array}{l} \ln\left(ASR\right)=\alpha \;+\;\beta x\;+\;\varepsilon \end{array}$$

x denotes the calendar year, β is the slope of the regression line, indicating the annual change, and ε is the residual error. The EAPC and its 95% confidence interval (CI) were derived as follows:


$$\begin{array}{l} EAPC=100\;\times \left(e^{\beta }-1\right) \end{array}$$

An increasing trend was inferred if the 95% CI of EAPC > 0. A decreasing trend was inferred if the 95% CI of EAPC < 0. A stable trend was concluded if the 95% CI included 0. The 95% CI was estimated using the linear regression model and propagated to the EAPC calculation.

All statistical analyses were conducted using R software (version 4.3.2). Data preprocessing and management were performed using the tidyverse and dplyr packages. Predictive modeling was carried out using the BAPC, INLA, and nordpred packages. Data visualization was performed using ggplot2. All statistical tests were two-sided, and a *p* < 0.05 was considered statistically significant.

## 3 Results

### 3.1 Global trends of nasopharyngeal cancer attributable to smoking and high alcohol use in older adults

Between 1990 and 2021, global age-standardized death rates (ASDR) attributable to smoking and high alcohol use exhibited significant declines. The ASDR for smoking decreased from 28.64 per 100,000 population (95% UI: 21.24–36.8) in 1990 to 16.35 (95% UI: 11.75–21.4) in 2021, corresponding to an EAPC of −2.11 (95% confidence interval (CI): −2.34 to −1.89). Similarly, the ASDR for high alcohol use declined from 22.67 (95% UI: 15.97–29.32) to 15.06 (95% UI: 10.73–19.52), with an EAPC of −1.58 (95% CI: −1.85 to −1.31). For high alcohol use, downward trends were observed across all SDI categories except low-middle SDI regions, which experienced a significant increase (EAPC: 1.01, 95% CI: 0.77–1.26), and low SDI regions, where a non-significant rise occurred (EAPC: 0.17, 95% CI: −0.11–0.45).

East Asia had the highest baseline ASDR for both smoking (86.85 per 100,000; 95% UI: 60.94–117.13) and high alcohol use (70.48; 95% UI: 47.29–96.16) in 1990. By 2021, this region achieved substantial reductions, with EAPCs of −2.89 (95% CI: −3.21 to −2.58) and −2.86 (95% CI: −3.23 to −2.50), respectively (2, 3). Australasia recorded the most rapid decline in smoking-related ASDR (EAPC: −5.02, 95% CI: −5.11 to −4.92) alongside a notable reduction in high alcohol use (EAPC: −2.39, 95% CI: −2.51 to −2.27). High–income North America (smoking EAPC: −2.71, 95% CI: −2.79 to −2.62; alcohol EAPC: −0.85, 95% CI: −0.97 to −0.73) and Western Europe (smoking EAPC: −3.11, 95% CI: −3.20 to −3.03; alcohol EAPC: −2.14, 95% CI: −2.22 to −2.07) also demonstrated consistent declines in both risk factors.

Conversely, Southeast Asia exhibited an upward trend in high alcohol use, with ASDR increasing from 11.19 to 21.22 per 100,000 (EAPC: +2.27, 95% CI: 2.15–2.39), despite reductions in smoking-related mortality (EAPC: −1.30, 95% CI: −1.39 to −1.22). This upward trajectory in alcohol-attributable NPC burden may be linked to region-specific socio-cultural practices and weak enforcement of alcohol control policies. For instance, in Vietnam and Indonesia, traditional consumption of rice-based spirits (in Vietnam) is deeply embedded in social rituals, while lax regulations on alcohol advertising have normalized heavy drinking among older males (20). Furthermore, alcohol taxation rates in Southeast Asia remain the lowest globally (e.g., 20% excise tax in Thailand vs. 70% in Australia), failing to curb affordability despite rising disposable incomes. Public health surveys indicate that only 35% of alcohol control laws in this region are fully implemented, with limited restrictions on sales to minors. Similar increases were observed in the Caribbean (EAPC: +1.04, 95% CI: 0.89–1.20) and Central Asia (EAPC: +0.83, 95% CI: 0.58–1.08). Central and Eastern Europe showed minimal changes in smoking-related ASDR (e.g., Central Europe EAPC: −0.19, 95% CI: −0.57–0.19) and only marginal fluctuations in alcohol-attributable deaths (e.g., Eastern Europe EAPC: −0.37, 95% CI: −0.72 to −0.01).

Sub-Saharan Africa displayed heterogeneous trends. Smoking-related ASDR declined across all subregions, though the magnitude varied substantially (EAPC range: −1.97, 95% CI: −2.10 to −1.85 in Western Sub-Saharan Africa to −0.47, 95% CI: −0.73 to −0.22 in Central Sub-Saharan Africa). High alcohol use remained stable or decreased slightly, with Southern Sub-Saharan Africa reporting the highest absolute ASDR in 2021 (11.73 per 100,000; EAPC: −0.53, 95% CI: −0.78 to −0.28).

**Figure 1:**
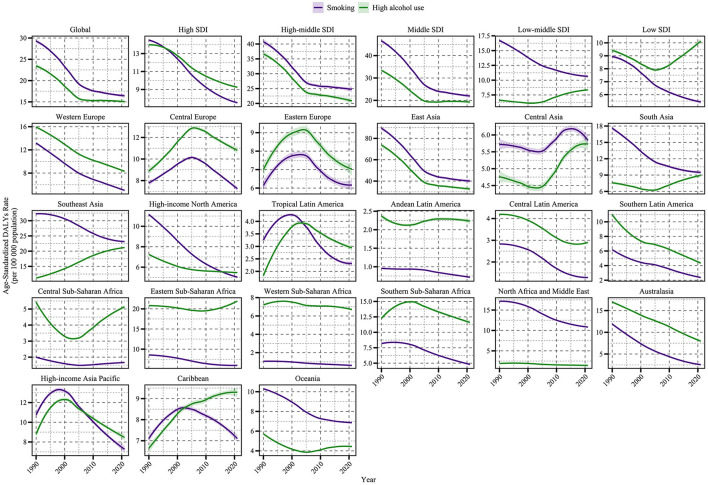
Time trends of age-standardized DALY rate (per 100,000 population) for NPC attributable to behavioral factors from 1990 to 2021, globally, across five SDI regions and in GBD regions. The figure illustrates the contributions of smoking (purple line) and high alcohol use (green line) to NPC DALYs.

**Figure 2:**
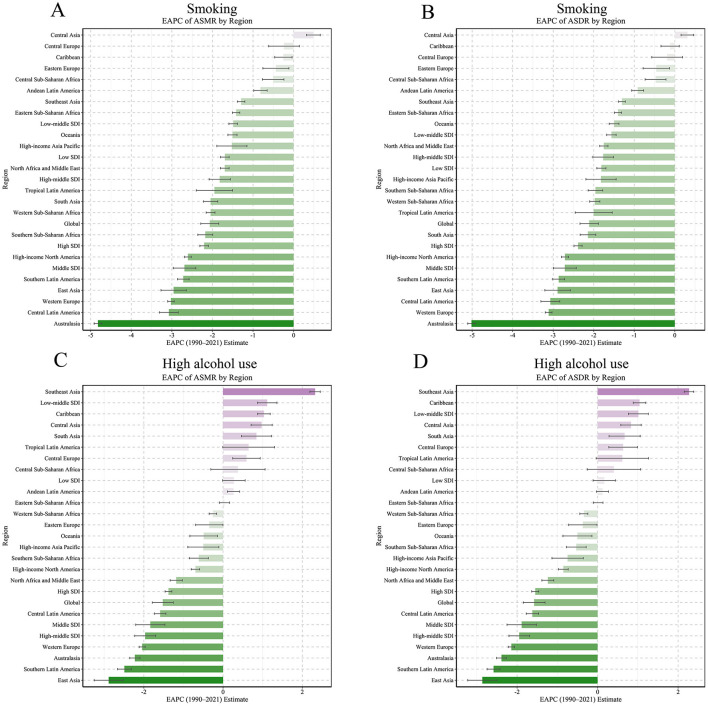
EAPC values of ASMR and ASDR (per 100,000 population) for NPC attributable to behavioral factors in 21 GBD regions (1990–2021). **(A)** EAPC of ASMR by region for smoking. **(B)** EAPC of ASDR by region for smoking. **(C)** EAPC of ASMR by region for high alcohol use. **(D)** EAPC of ASDR by region for high alcohol use.

NPC burden attributable to behavioral risks varied markedly by SDI (Figures 1, 2, Supplementary Table S1). SDI-stratified burden patterns (Supplementary Table S1) highlighted two critical anomalies: (1) despite overall declines, low-middle SDI regions exhibited rising alcohol-attributable ASDR (EAPC: +1.01), driven by unregulated markets in Southeast Asia; and (2) middle SDI regions paradoxically combined the steepest smoking declines (−2.71 EAPC) with the highest absolute rates (ASDR: 25.3 per 100,000), reflecting lagged policy responses to earlier tobacco epidemics. Middle and high-middle SDI regions consistently exhibited the highest ASDR and ASRs throughout the study period. While ASRs declined in almost all SDI categories, there was slower progress and an increase in low and medium SDI areas, particularly for alcohol-attributable mortality (low and medium SDI EAPC: +1.01, 95% CI: 0.77–1.26; low SDI EAPC: +0.17, 95% CI: −0.11–0.45), which highlights the fact that older adults experience NPC's persistent health disparities.

### 3.2 Regional patterns and temporal changes in nasopharyngeal cancer burden of older adults

**Figure 3:**
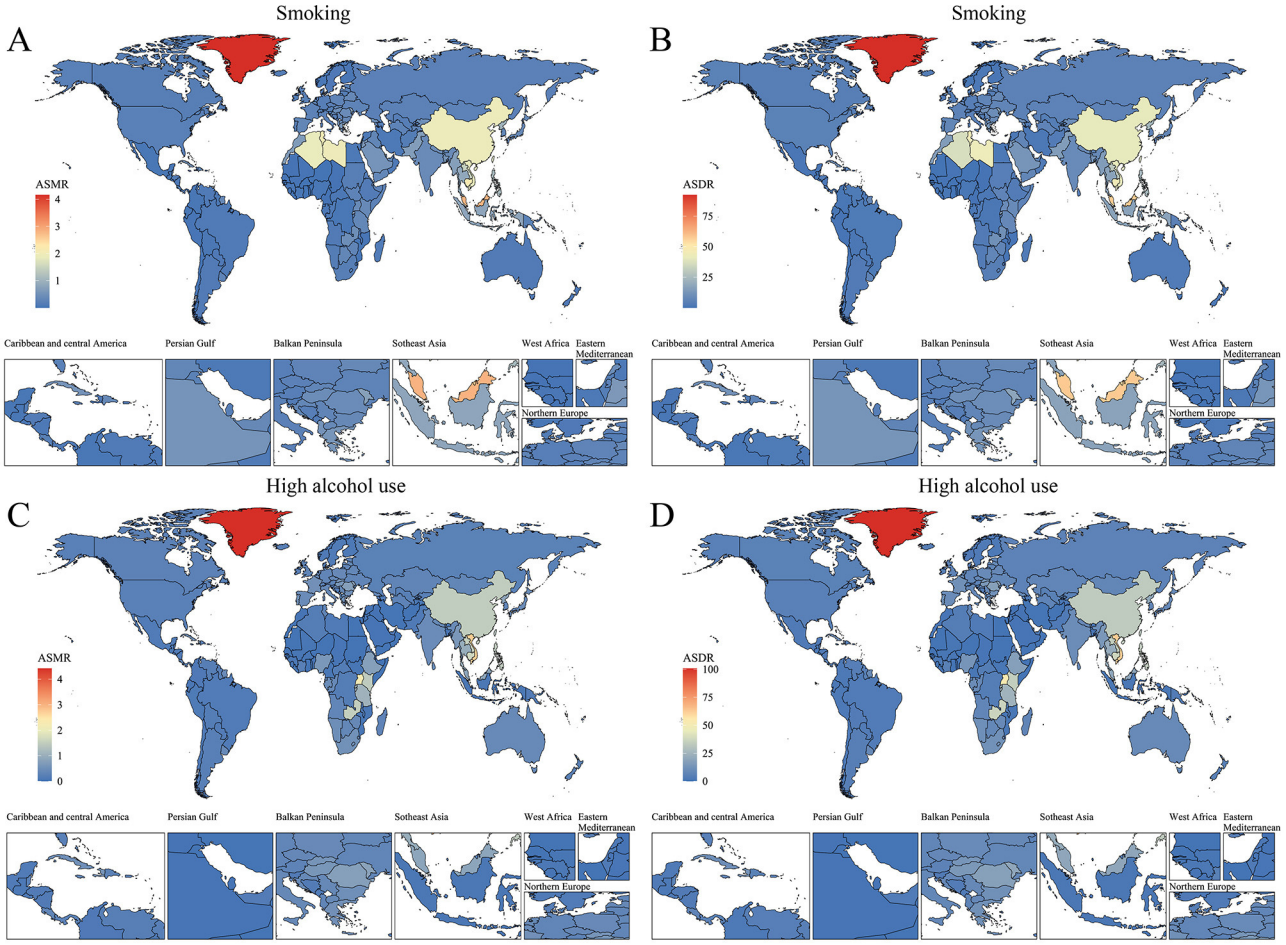
Spatial distribution of ASMR and ASDR (per 100,000 population) for NPC attributable to behavioral factors in 2021. **(A)** ASMR by region for smoking. **(B)** ASDR by region for smoking. **(C)** ASMR by region for high alcohol use. **(D)** ASDR by region for high alcohol use.

**Figure 4:**
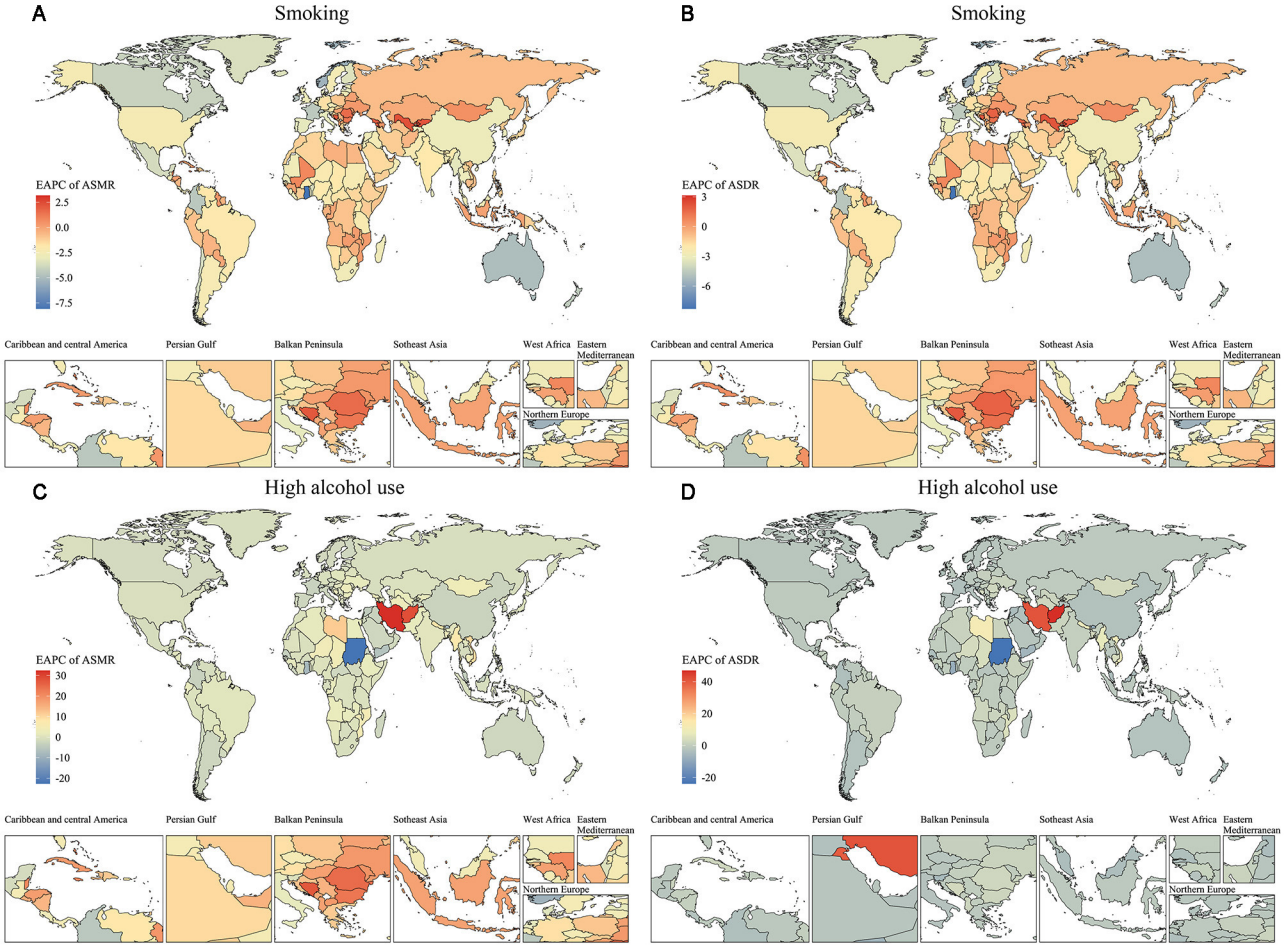
Spatial distribution of EAPC for ASMR and ASDR (per 100,000 population) of nasopharyngeal cancer attributable to behavioral factors from 1990 to 2021. **(A)** EAPC of ASMR by region for smoking. **(B)** EAPC of ASDR by region for smoking. **(C)** EAPC of ASMR by region for high alcohol use. **(D)** EAPC of ASDR by region for high alcohol use.

Sub-Saharan Africa and South Asia showed more modest reductions or increases in alcohol-attributable burden. Regional analyses revealed marked differences in the temporal evolution of NPC burden for older adults. In 2021, East Asia had the highest age-standardized DALY rate of smoking-attributable NPC for older adults at 40.07 (95% UI: 27.13–55.27) per 100,000, followed by middle SDI regions (Figure 3). For high alcohol use, Southeast Asia (21) exhibited a rising trend with an EAPC of 2.27 (95% CI: 2.15 to 2.39). In contrast, regions such as Western Europe and Australasia showed rapid declines in ASMR and ASDR for both risk factors. The contrasting trends between Southeast Asia and high-income regions (e.g., Australasia) highlight the critical role of policy enforcement. While Australia achieved a 40% reduction in alcohol-related harms through strict advertising bans and volumetric taxation (22), Southeast Asian nations face systemic challenges. For example, in the Philippines, alcohol companies sponsor local festivals, directly targeting older adult populations through culturally resonant campaigns. Such practices, coupled with minimal public awareness programs, exacerbate high-risk behaviors among older adults. At the national level, China, Indonesia, and the Philippines recorded the highest DALY numbers for smoking-related NPC, while Vietnam and Thailand also showed considerable alcohol-related burdens (Figure 4). Countries with the highest ASMR and ASDR in 2021 include China (smoking) and Vietnam (alcohol use). The steepest declines occurred in Australia and New Zealand (EAPC < -4 for smoking), whereas several Southeast Asian countries exhibited increasing trends in alcohol-attributable NPC burden.

### 3.3 Gender and age disparities in nasopharyngeal cancer mortality and DALYs attributable to behavioral factors

Age-specific patterns revealed that older males aged 60–79 years faced the highest relative risks (11, 23). The decline in age-specific mortality sex ratios among the oldest age groups (80+ years), such as a reduction from 150:1 to 100:1 in East Asia, may reflect survivor bias (selective mortality of high-risk individuals) or cumulative passive smoking exposure in older adult females, particularly in low-resource settings. Sex disparities were starkly pronounced, driven by both biological and behavioral factors.

**Figure 5:**
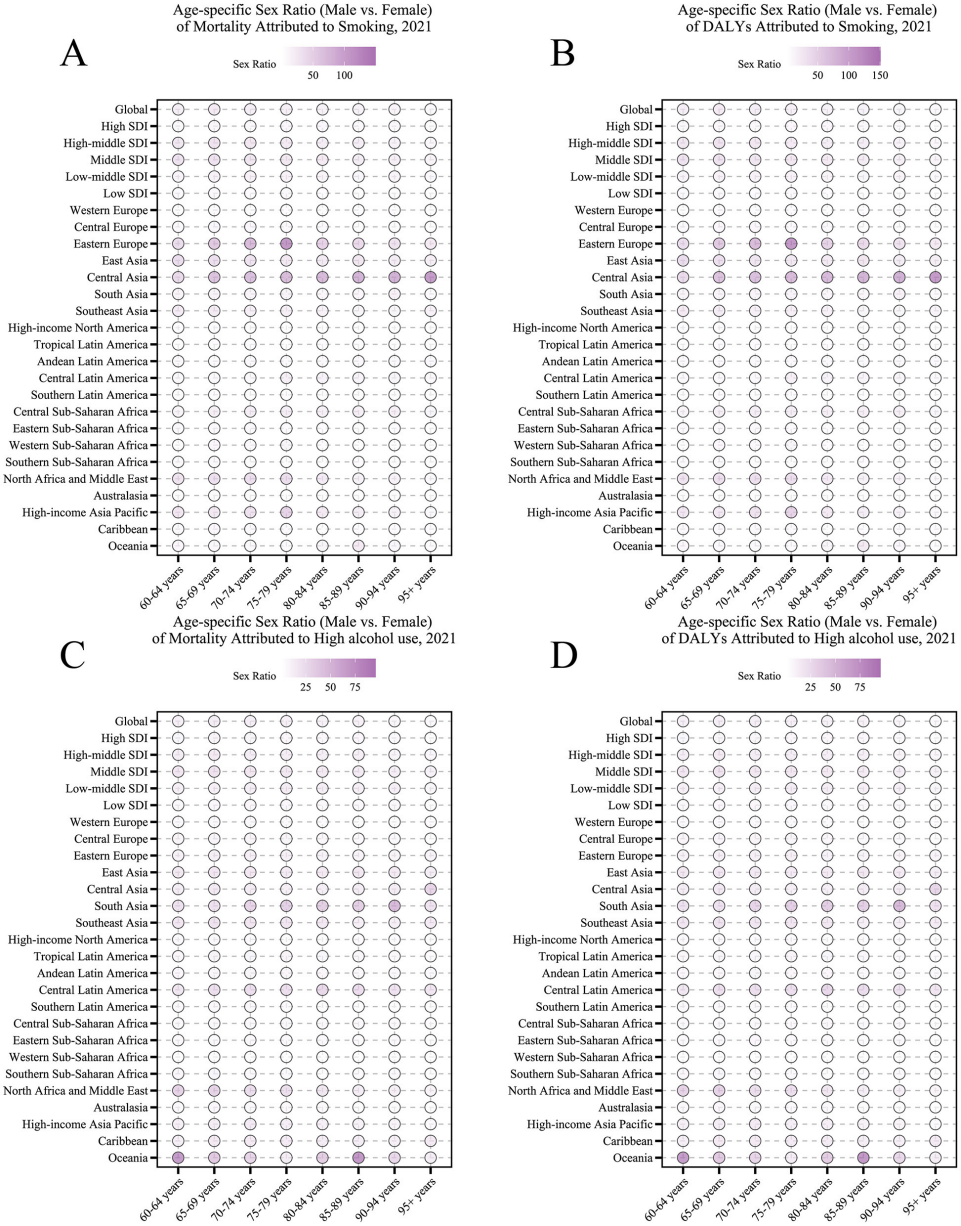
Age-specific sex ratio (older male vs. female) of nasopharyngeal cancer attributable to behavioral factors in 2021. **(A)** Age-specific sex ratio of mortality due to smoking. **(B)** Age-specific sex ratio of DALYs due to smoking. **(C)** Age-specific sex ratio of mortality due to high alcohol use. **(D)** Age-specific sex ratio of DALYs due to high alcohol use.

For smoking-related outcomes, the extreme age-specific DALYs sex ratio of 150:1 in Central Asia correlated with high older male smoking prevalence (>60%) and synergistic interactions with endemic EBV infection. In high alcohol use regions, age-specific mortality sex ratios reached 75:1 in South Asia and Central Latin America, aligning with cultural norms of heavy spirit consumption among older males (Figures 5A, B). Notably, the age-specific DALYs sex ratio for alcohol-related conditions peaked at 50:1 in Central Latin America's 70–79-year age group, underscoring the long-term disability burden from chronic alcohol use (Figures 5C, D).

**Figure 6:**
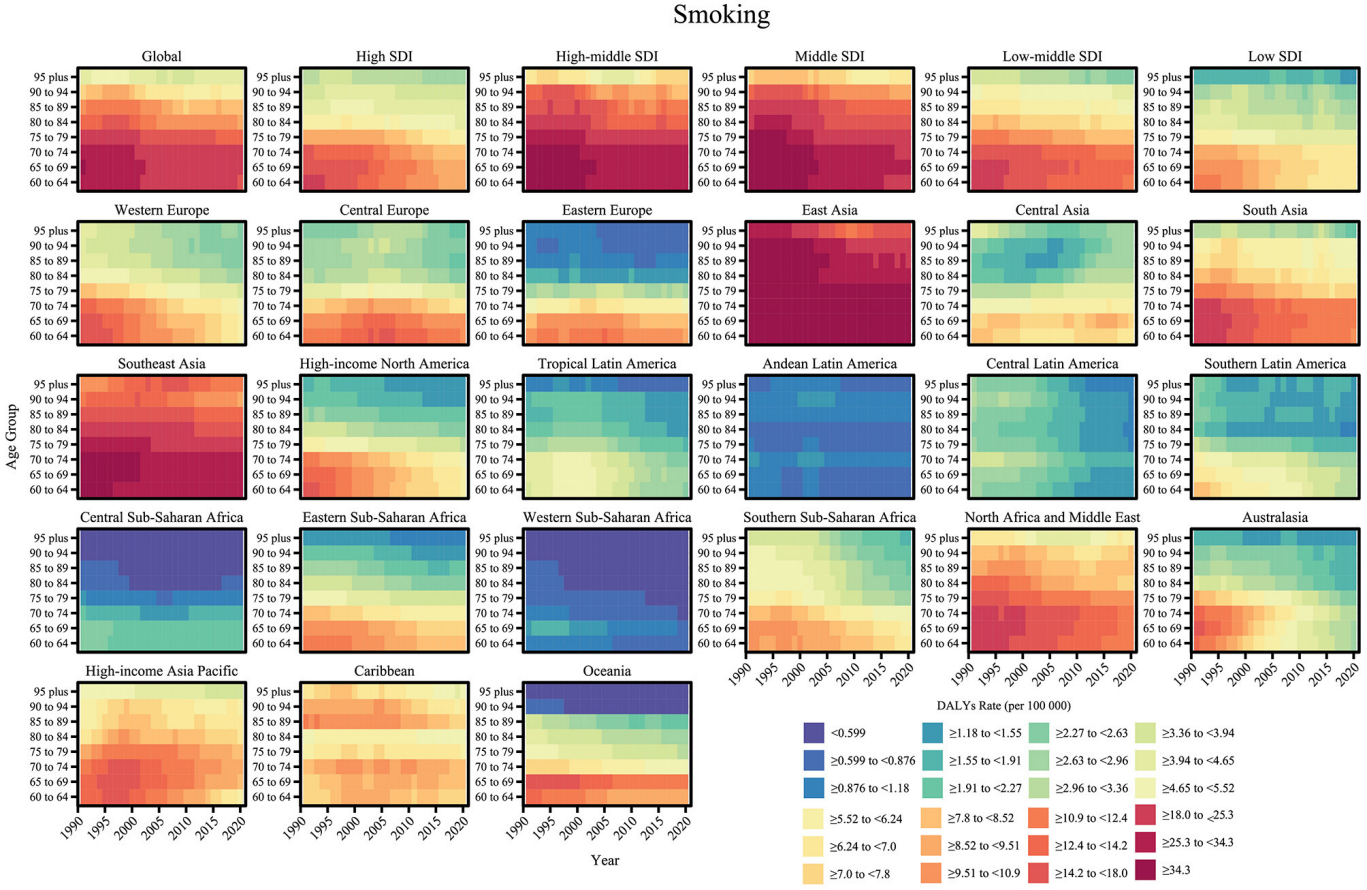
Age-specific trends of DALY rate (per 100,000 population) for nasopharyngeal cancer attributable to smoking from 1990 to 2021.

**Figure 7:**
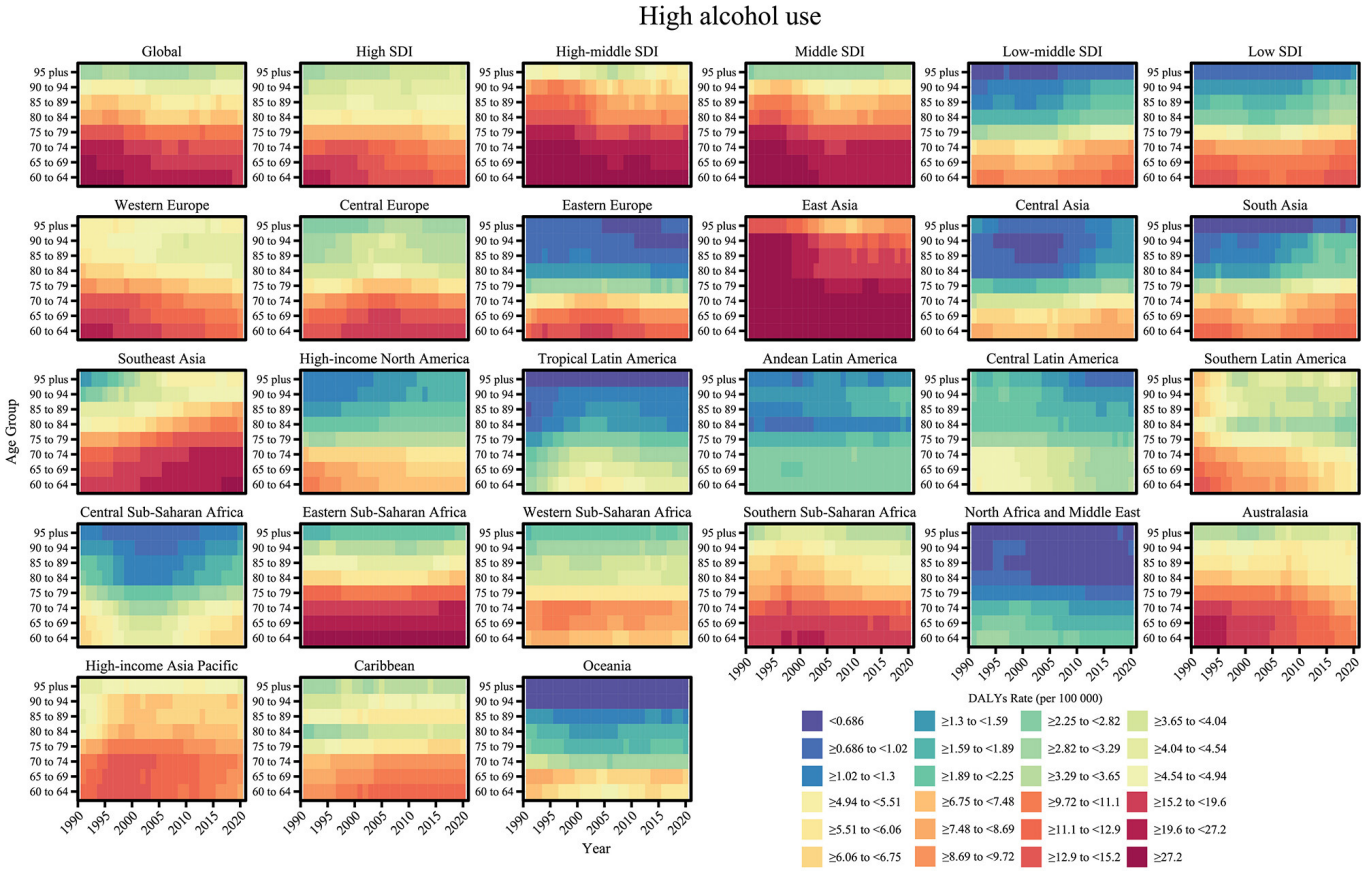
Age-specific trends of DALY rate (per 100,000 population) for nasopharyngeal cancer attributable to high alcohol use from 1990 to 2021.

From 1990 to 2021, the impact of smoking and high alcohol use on nasopharyngeal cancer-related DALYs showed significant age dependence, regional heterogeneity, and temporal dynamics. In terms of age trends, the rate of DALY due to smoking decreased consistently with age. The DALY rate due to high alcohol use, although lower overall, showed a steeper age gradient, with an accelerated decline especially in the 80–84 age group, suggesting that the synergistic effect of alcohol exposure and the aging process may reduce the risk of the disease. In terms of temporal trends, smoking- and alcohol-related DALY rates have declined globally, mainly attributable to strict tobacco and alcohol control policies and the spread of early screening programs in high SDI regions (e.g., Western Europe and North America), where the burden in the 60–74-year-old age group has been reduced by 15–20% since 2000. Regarding regional differences, the burden of smoking is most prominent in middle SDI regions, with DALY rates of 18.0–25.3 per 100,000 for 60–64-year-olds, while the burden for the same age group in low SDI regions is only 5.52–8.52 per 100,000. The high-risk regions for excessive alcohol and tobacco use were East and Southeast Asia, with DALY rates of 19.6–27.2 and 25.3–34.3 per 100,000 people aged 60–64 years, which may be synergistically associated with dietary risks such as drinking culture and salted fish intake, while the low-risk regions were Andean Latin America with DALY rate of < 1/100,000 for people aged 60–64 years (Figures 6, 7).

Policy-level strategies need to be tailored to high-risk age groups and regions: in the high-middle SDI region, nasopharyngeal cancer screening should be intensified in the older adults to reduce late diagnosis, and the spread of risk factors should be curbed through tobacco and alcohol tax reforms, primary healthcare capacity enhancement, and public education. In addition, the Global Coordinating Mechanism (GCM) needs to improve the DALY attribution methodology to clarify the interaction of smoking, alcohol, and other factors (e.g., EBV and genetic predisposition).

### 3.4 Global trends in risk factor attributable burden of older adults (1990–2021)

**Figure 8:**
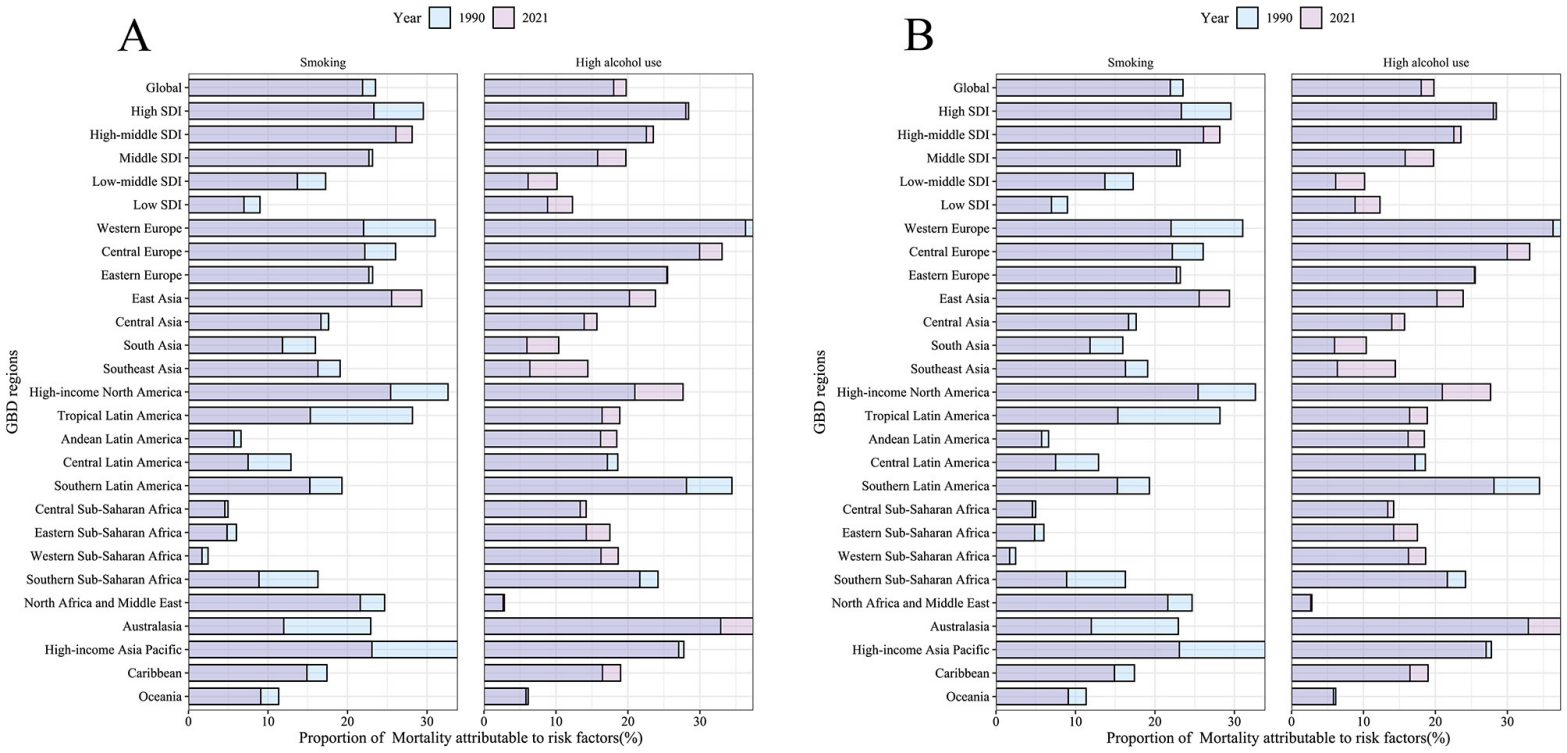
The proportion of ASMR and ASDR for nasopharyngeal cancer attributable to behavioral risk factors in 1990 and 2021. **(A)** The proportion of ASMR due to smoking and high alcohol use (1990 vs. 2021). **(B)** Proportion of ASDR due to smoking and high alcohol use (1990 vs. 2021). The figure is categorized by global, various SDI regions, and GBD regions.

Based on the analysis of DALYs and mortality data of older adults, the burden of disease associated with smoking decreased globally from 23.53% in 1990 to 21.94% in 2021, and the proportion of deaths decreased from 23.52% to 21.89%, indicating that tobacco control measures have been somewhat effective; however, the burden of disease associated with high alcohol use increased from 18.03% to 19.79%. However, the burden of disease for high alcohol use increased from 18.03% to 19.79%, and the mortality rate increased from 18.02% to 19.76%, highlighting the increase in alcohol-related health risks (Figure 8). Regional differences were significant: while smoking DALYs decreased significantly from 29.55% to 23.32% and mortality rates from 29.53% to 23.32% in high SDI regions, reflecting their stringent tobacco control policies, high alcohol use DALYs increased only marginally from 28.05% to 28.46%, and mortality rates decreased slightly from 28.05% to 28.43%. The opposite trend was observed in high-moderate SDI regions, where smoking DALYs increased from 26.10% to 28.16% and mortality rates from 26.09% to 28.13%, and in low SDI regions, where smoking DALYs declined from 8.99% to 6.98% but high alcohol use increased from 8.83% to 12.29%, with a corresponding increase in mortality rates from 8.83% to 12.29%, indicating a worsening of the alcohol abuse problem in low-income countries.

In East Asia, smoking DALYs increased from 25.56% to 29.37%, and mortality rates increased from 25.55% to 29.34%; in Western Europe, smoking DALYs decreased from 31.05% to 22.02%, and high alcohol use DALYs decreased slightly from 37.42% to 36.35%, with a corresponding percentage of mortality rates decreasing from 37.41% to 36.33%. North Africa and the Middle East had very low rates of high-alcohol use (DALYs increased slightly from 2.66% to 2.83%, and mortality rates remained stable); Australia's high-alcohol use DALYs spiked from 32.92% to 37.43%, and mortality rates increased from 32.89% to 37.41%. Tropical Latin America smoking DALYs plummeted from 28.18% to 15.33% and mortality from 28.17% to 15.33%, while Andean Latin America high-alcohol use DALYs rose from 16.20% to 18.46% and mortality from 16.20% to 18.45%, indicating region-specific risks. Overall, tobacco control is effective, but alcohol problems persist in high-income regions, middle-income regions face a double challenge, and low-income regions are at increased risk of alcohol use and require differentiated policy interventions.

**Figure 9:**
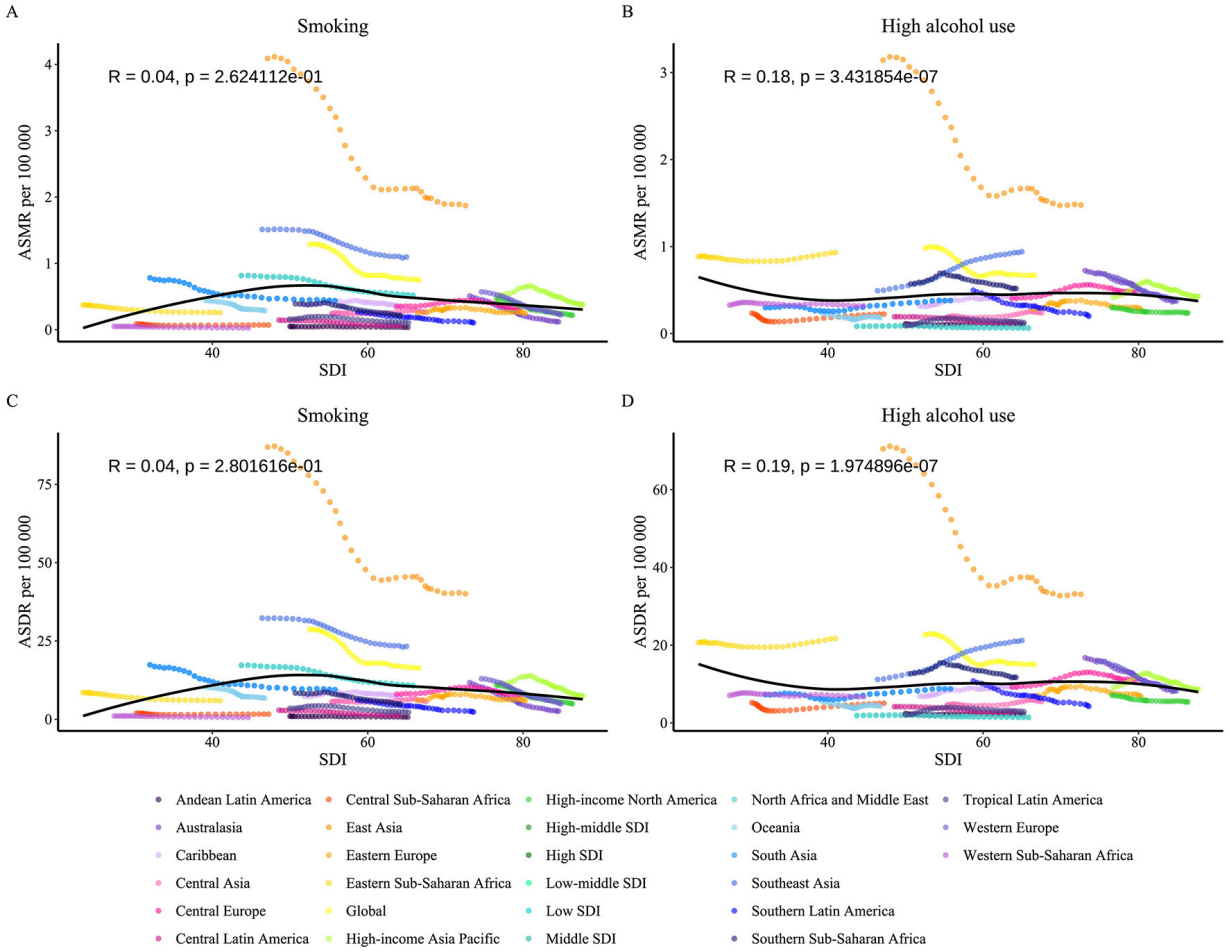
From 1990 to 2021, the age-standardized mortality rate and DALY rate of nasopharyngeal cancer caused by behavioral factors in 21 GBD regions were classified by the SDI. The expected values based on SDI and burden indicators for all locations are represented by black lines. Statistical test results are shown at the top left of each subplot. **(A)** ASMR for smoking. **(B)** ASMR for high alcohol use. **(C)** ASDR for smoking. **(D)** ASDR for high alcohol use. ASMR, Age-Standardized Mortality Rate; ASDR, Age-Standardized DALY Rate; DALY, Disability-Adjusted Life Years; SDI, Sociodemographic Index.

We found that the associations of ASMR and ASDR with SDI for NPC varied according to behavioral risk factors. Smoking-related burden (ASMR: R = 0.04, *p* = 0.262; ASDR: R = 0.04, *p* = 0.280) (Figures 9A, C) was not significantly associated with SDI, suggesting that the impact of smoking on NPC may be driven more by non-socioeconomic factors such as heredity or environmental exposure. In contrast, high alcohol use-related burden showed a weak but significant positive correlation with SDI (ASMR: R = 0.18, *p* = 3.43 × 10-7; ASDR: R = 0.19, *p* = 1.97 × 10-7) (Figures 9B, D) in moderate SDI regions, possibly reflecting the tension between increased alcohol consumption and lagging public health interventions in middle-income countries. NPC burden significantly deviated from expected values in sub-Saharan East Africa (low SDI), Southeast Asia (low-middle SDI), and East Asia (high-middle SDI), highlighting the complexity of region-specific risk mechanisms. The high burden in East Asia was strongly associated with EBV infection, HLA gene polymorphisms, and a traditional high-salt diet (9, 24). In contrast, in Southeast Asia, it was associated with betel nut chewing, exposure to industrial pollutants, and delayed healthcare-seeking behavior. Sub-Saharan East Africa has an unusually high burden due to the high prevalence of EBV (>90%), HIV co-infection, and lack of radiotherapy resources (e.g., only one radiotherapy unit in the whole country in Uganda). Our study reveals the non-linear character of the relationship between socioeconomic indices and disease burden, emphasizing the centrality of multidimensional risk coevolution mechanisms in cancer prevention and control.

### 3.5 Global distribution and hotspot evolution

In 2050, projections incorporated three key assumptions: population age structures were projected using United Nations probabilistic forecasts (2022 Revision), assuming medium fertility and mortality variants. Age-sex-specific smoking and alcohol use prevalence was extrapolated based on 2010–2021 trajectories, assuming no major policy changes (“policy stagnation” scenario). Age-standardized mortality-to-incidence ratios were held constant at 2021 levels.

**Figure 10:**
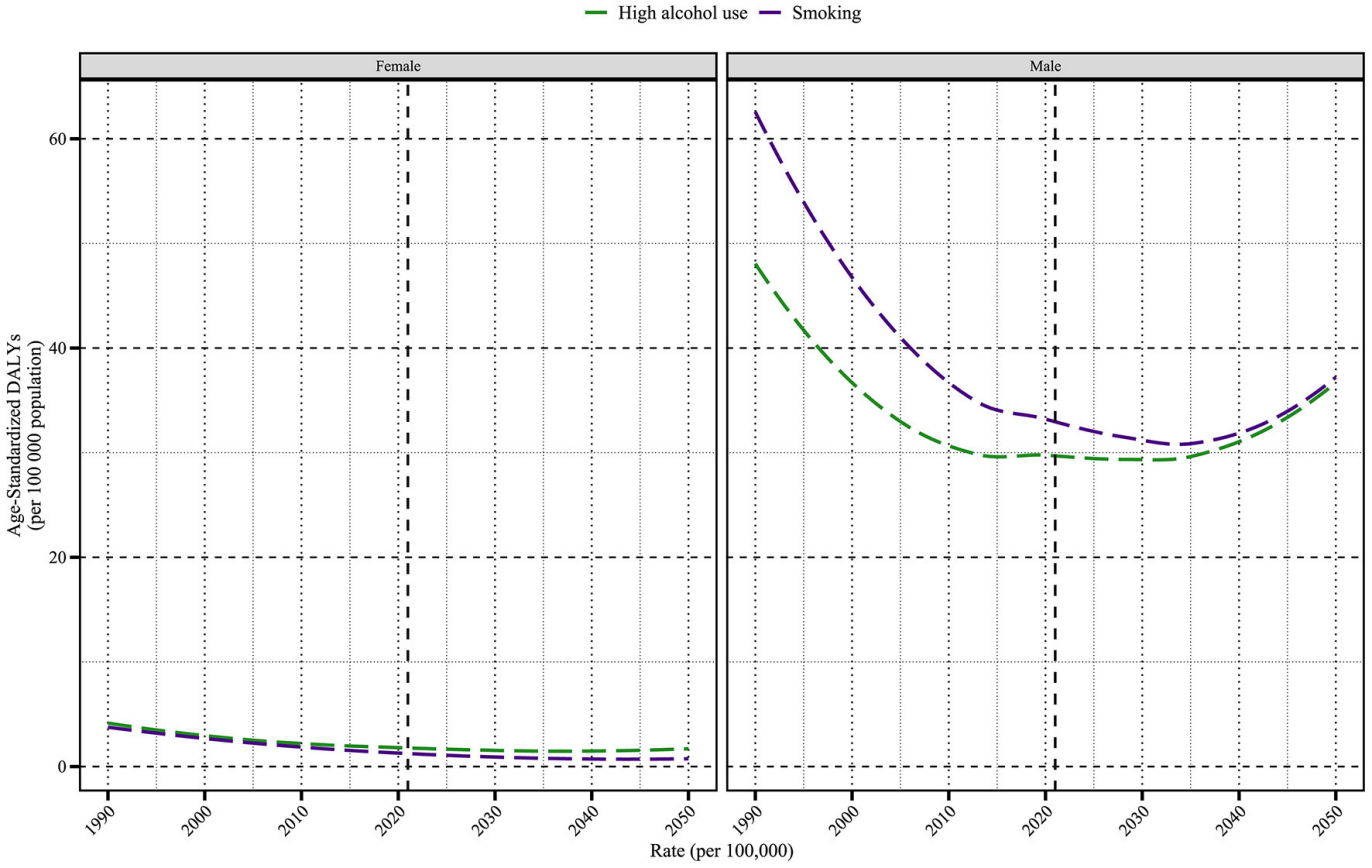
The projected trends of age-standardized DALY rate (per 100,000 population) for nasopharyngeal cancer attributable to behavioral factors from 1990 to 2050. The figure illustrates the trends of DALY rates due to smoking and high alcohol use in female and male populations. DALY, Disability-Adjusted Life Year.

The DALYs per 100,000 population outcomes due to smoking and high alcohol use for older males and females through 2050 show significant differences. Smoking-related DALYs were 39.31 per 100,000 for older males and 0.77 per 100,000 for older females, ~51 times higher for older males than for older females, and high alcohol use-related DALYs were 38.66 per 100,000 for older males and 1.80 per 100,000 for older female, 21.5 times higher for older male than for older female. The health burden of smoking was slightly higher than that of high alcohol use among older males (39.31 vs. 38.66 per 100,000), while the burden of high alcohol use was significantly higher among older females (1.80 vs. 0.77 per 100,000). Long-term trend analyses showed that smoking DALYs among older males have continued to decline since 1990 (59.50 per 100,000), while high alcohol use showed a fluctuating trend of decreasing and then increasing, eventually approaching the level of smoking; DALYs among older females were lower overall in both risk categories, but high alcohol use was projected to increase in 2050 compared with the previous period (Figure 10). The above results suggest that older males' health burden is still dominated by smoking and high alcohol use, and intensified targeted interventions are needed, while the potential increase in the risk of high alcohol use in older females may be associated with changes in behavioral patterns, and enhanced surveillance and early prevention are needed.

## 4 Discussion

This study comprehensively analyzed the global, regional, and national burden of older adults' NPC attributable to smoking and high alcohol use from 1990 to 2021 and projected trends to 2050. To the best of our knowledge, this is the first study to simultaneously evaluate the long-term spatiotemporal trends, sociodemographic patterns, and sex disparities of NPC linked to these two major behavioral risk factors, using data from the Global Burden of Disease Study. The concentration of NPC burden in middle and high-middle SDI regions reflects a paradoxical interplay of rising risk exposure (e.g., delayed tobacco control adoption) and suboptimal healthcare access. For instance, middle SDI nations (e.g., China) experienced rapid economic growth accompanied by surging alcohol consumption, while high-middle SDI regions (e.g., Southeast Asia) lagged in enforcing advertising bans. In contrast, low SDI settings faced dual challenges of limited screening infrastructure and persistent EBV endemicity. Our findings extend prior GBD analyses in three key dimensions: First, we quantify the attributable burden of smoking and alcohol use, two understudied yet modifiable factors. Second, our stratification by SDI quintiles and sex uncovers previously masked inequities, such as the 51-fold higher smoking-related DALY rates in older males vs. females in LMICs—a disparity unaddressed in the existing literature.

Globally, the ASRs of DALYs and deaths showed significant downward trends, particularly for smoking. Smoking consistently contributed a higher burden than high alcohol use, both in terms of DALYs and deaths. Nevertheless, the gap between these two risk factors is narrowing in several regions, particularly in LMICs, where alcohol use is on the rise.

Marked regional heterogeneity was observed. East Asia remained the epicenter of NPC burden, with the highest ASRs and absolute numbers of DALYs for both risk factors, largely reflecting the endemicity of EBV, high smoking prevalence, and environmental co-exposures. Encouragingly, East Asia also showed one of the most pronounced decreases in ASRs, suggesting effective tobacco control policies and improved health awareness. By SDI level, middle and high-middle SDI regions bore a disproportionately high burden, consistent with higher risk exposure and suboptimal health system performance. Conversely, high SDI regions, particularly Australasia and Western Europe, showed the steepest declines in ASRs and the most effective risk mitigation, likely due to comprehensive smoking cessation programs, alcohol regulation policies, and better access to early diagnosis and care.

A prominent and persistent older male predominance in NPC burden attributable to both smoking and alcohol was evident across all regions. This is likely driven by a higher prevalence of risk behaviors among older males, biological differences in carcinogen metabolism, and delayed health-seeking behavior. The pronounced male predominance in NPC burden may stem from synergistic biological and behavioral mechanisms. Biologically, androgen receptor signaling has been shown to enhance EBV replication and promote nasopharyngeal epithelial cell proliferation, potentially amplifying carcinogenic effects in males. Additionally, sex-specific differences in carcinogen metabolism, such as lower expression of detoxifying enzymes (e.g., glutathione S-transferase) in males (25), may increase susceptibility to tobacco-derived nitrosamines. Behaviorally, cultural norms in many regions normalize heavy smoking and alcohol consumption among older males. For instance, in East Asia, 68% of males aged ≥60 years report daily smoking compared to 3% of females, while alcohol use prevalence is 4.5-fold higher in males. Occupational exposures (e.g., formaldehyde in male-dominated industries) and delayed healthcare-seeking behavior (e.g., 30% lower clinic attendance rates in males with early symptoms) further exacerbate disparities. Although some high SDI regions showed a slight narrowing of the older male-to-female ratio, the global trend remains largely unchanged, calling for more gender-sensitive prevention strategies. The significant decreases in ASRs and negative EAPC in most regions reflect the success of anti-smoking campaigns, alcohol taxation policies, and enhanced screening and healthcare access. To address the disproportionate burden among older males, gender-responsive policies must be prioritized. For instance, workplace-based NPC screening programs in male-dominated industries (e.g., mining and construction) could leverage routine occupational health checks. In South Africa, integrating EBV serology testing into silica dust exposure assessments increased early NPC detection by 35% among male miners aged ≥60 years. These approaches align prevention with masculine social roles, reframing healthcare engagement as a communal responsibility rather than individual weakness. However, regions with increasing or stable trends—such as Southeast Asia, Central Asia, and parts of Sub-Saharan Africa—require urgent public health interventions to halt and reverse the rising trajectories.

Our projections for 2050 suggest that while the global age-standardized burden will likely continue to decline, absolute DALYs and deaths are projected to increase, particularly in LMICs. This underscores the need for context-specific, long-term prevention strategies, including behavioral change campaigns, environmental regulation, and health system strengthening. A major strength of this study is the use of the GBD 2021 database (24, 26), enabling a globally comparable, comprehensive assessment over three decades. The inclusion of both smoking and alcohol allows for an integrated view of behavioral risk contributions.

However, some limitations should be acknowledged. First, GBD estimates rely on modeled data, especially in data-scarce regions, which may introduce uncertainty. Second, the synergistic effects of smoking and alcohol, as well as their interaction with EBV infection, were not separately quantified. Regional cultural and policy differences affecting behavior change were not incorporated into the projections. Finally, our analysis did not account for emerging risk factors that may confound the observed trends. For instance, the rapid adoption of vaping in high SDI regions could alter smoking-related risk profiles, as early evidence suggests that dual use of cigarettes and e-cigarettes may exacerbate upper respiratory tract inflammation and EBV reactivation (27).

Our findings highlight the continuing global burden of NPC attributable to smoking and alcohol, with substantial disparities across regions, SDI levels, and sexes. Prevention strategies must be tailored to local contexts and should include (1) strengthening tobacco and alcohol control laws, (2) expanding public education and behavioral interventions, (3) targeting high-risk older adult populations, especially older males in LMICs, and (4) investing in early detection and cancer care infrastructure. On-time policy action is essential to reduce the future burden and to achieve the NPC-related targets of global non-communicable disease prevention frameworks. Beyond conventional policies, emerging technologies and community-based approaches offer novel pathways for risk reduction. For instance, mobile health (mHealth) platforms—such as AI-driven chatbots delivering personalized smoking cessation support—have shown efficacy in rural China, achieving a 22% quit rate among older adult smokers through daily behavioral nudges. Similarly, community health worker networks in Sub-Saharan Africa integrate NPC screening with HIV/AIDS programs, leveraging existing trust to improve early detection rates by 30% in high-risk populations. At the systemic level, geospatial analytics could prioritize regions with overlapping high alcohol use and EBV prevalence for targeted vaccine deployment.

## 5 Conclusion

This comprehensive global assessment of nasopharyngeal cancer caused by tobacco and alcohol use among older adults reveals progress and continuing challenges. The decline in ASRs demonstrates the effectiveness of tobacco control policies and alcohol reduction strategies, particularly in high SDI countries. However, the growing burden in some low and medium SDI areas calls for urgent policy interventions and prevention strategies. Health education, early screening, and culturally sensitive interventions are essential to reduce the projected burden by 2050. Our study highlights the need to tailor nasopharyngeal cancer prevention programs regionally based on observed trends and risk factor attribution.

## Data Availability

The original contributions presented in the study are included in the article/Supplementary material, further inquiries can be directed to the corresponding author.

## Supplementary material

Supplementary Table S1 ASMR and ASDR of NPC attributable to smoking and high alcohol use across SDI regions (1990–2021), with key trends highlighted.
